# Temporal Associations Between Social Activity and Mood, Fatigue, and Pain in Older Adults With HIV: An Ecological Momentary Assessment Study

**DOI:** 10.2196/mental.9802

**Published:** 2018-05-14

**Authors:** Emily W Paolillo, Bin Tang, Colin A Depp, Alexandra S Rooney, Florin Vaida, Christopher N Kaufmann, Brent T Mausbach, David J Moore, Raeanne C Moore

**Affiliations:** ^1^ San Diego State University/University of California San Diego Joint Doctoral Program in Clinical Psychology San Diego, CA United States; ^2^ Department of Psychiatry University of California, San Diego San Diego, CA United States; ^3^ VA San Diego Healthcare System San Diego, CA United States; ^4^ The Sam and Rose Stein Institute for Research on Aging University of California, San Diego San Diego, CA United States

**Keywords:** AIDS, ecological momentary assessment, social isolation, happiness, quality of life

## Abstract

**Background:**

Social isolation is associated with an increased risk for mental and physical health problems, especially among older persons living with HIV (PLWH). Thus, there is a need to better understand real-time temporal associations between social activity and mood- and health-related factors in this population to inform possible future interventions.

**Objective:**

This study aims to examine real-time relationships between social activity and mood, fatigue, and pain in a sample of older PLWH.

**Methods:**

A total of 20 older PLWH, recruited from the University of California, San Diego HIV Neurobehavioral Research Program in 2016, completed smartphone-based ecological momentary assessment (EMA) surveys 5 times per day for 1 week. Participants reported their current social activity (alone vs not alone and number of social interactions) and levels of mood (sadness, happiness, and stress), fatigue, and pain. Mixed-effects regression models were used to analyze concurrent and lagged associations among social activity, mood, fatigue, and pain.

**Results:**

Participants (mean age 58.8, SD 4.3 years) reported being alone 63% of the time, on average, (SD 31.5%) during waking hours. Being alone was related to lower concurrent happiness (beta=−.300; 95% CI −.525 to −.079; *P*=.008). In lagged analyses, social activity predicted higher levels of fatigue later in the day (beta=−1.089; 95% CI −1.780 to −0.396; *P*=.002), and higher pain levels predicted being alone in the morning with a reduced likelihood of being alone as the day progressed (odds ratio 0.945, 95% CI 0.901-0.992; *P*=.02).

**Conclusions:**

The use of EMA elucidated a high rate of time spent alone among older PLWH. Promoting social activity despite the presence of pain or fatigue may improve happiness and psychological well-being in this population.

## Introduction

Social isolation is a well-known risk factor for incident mental and physical health problems, including depression [1], distressing somatic symptoms (eg, pain and fatigue) [2], substance use [3], cognitive impairment [4], heart disease [5], and mortality [6]. In fact, meta-analytic work has shown that having social relationships is associated with reduced mortality risk comparable with that of well-known health factors such as not smoking (vs smoking), abstaining from alcohol (vs binge drinking), engaging in rigorous physical activity (vs low-to-no physical activity), and being lean (vs obese) [7]. Notably, however, the relationship between social activity and health is also reciprocal, such that chronic illnesses and diminished health can result in physical (eg, disability), emotional (eg, stigma and stress), and financial barriers to engagement in social activities, leading to social isolation [8].

Older persons living with HIV (PLWH) are a rapidly growing population of individuals who are at a significantly increased risk for social isolation compared with older persons without HIV [9]. In addition to social barriers that older HIV-uninfected adults often face (eg, retirement, hearing loss, and mobility impairment) [10], older PLWH face barriers to social support that are specifically related to HIV infection, including HIV-related stigma and discrimination [11], nondisclosure of HIV status [12], and loss of their social support network because of AIDS-related deaths [13]. Furthermore, these individuals also often have several medical comorbidities (eg, chronic diarrhea, lipodystrophy, cardiovascular disease, cancers, kidney and liver diseases, and osteoporosis) that can cause significant emotional distress, fatigue, and pain, leading to further social withdrawal [14-16]. Fatigue and pain are common somatic symptoms of HIV infection and can be critical barriers to engagement in social activity [17,18]. Results from a recent study conducted by Moore et al support findings of decreased social activity among older PLWH such that participants reported spending a majority of their sampled time at home, alone, and engaged in passive leisure activities (eg, watching television) [19]. Furthermore, older PLWH are more likely to live alone compared with both younger PLWH and their seronegative counterparts [9,12], greatly increasing the risk for social isolation.

In addition to the mental and physical health consequences of social isolation in the general population, older PLWH are also particularly vulnerable to worsening HIV disease progression without adequate social support [20]. Possible mechanisms for this include increased depression and reduced psychological well-being in older PLWH who are socially isolated [21], which in turn can contribute to decreased immune function and nonadherence to antiretroviral therapy medication [22,23]. Despite the increased risk for, and long-term detrimental consequences of, social isolation among older PLWH, there is a lack of research on the real-time temporal associations of social activity and mood- and health-related factors in this population.

Ecological momentary assessment (EMA) is a data collection method that has potential to help fill a gap in research on social activity among older PLWH. EMA is a highly feasible, valid, and reliable way to obtain real-world, real-time evaluations of various behaviors (eg, social activity) and experiences (eg, mood, fatigue, and pain) in a range of clinical and nonclinical samples, including older adults [19,24,25]. Thus, EMA can allow us to examine the temporal dynamics of social activity, in particular whether social activity predicts mood- and health-related factors (ie, fatigue and pain) or vice versa. Previous studies suggest that these relationships are often reciprocal and cyclical—ie, levels of social activity influence mental and physical health, and mental and physical health simultaneously influence levels of social activity, creating a potential downward spiral of decreasing social activity and worsening health outcomes [26]; however, such real-time relationships have yet to be examined among older PLWH.

Therefore, this study aims to use EMA to examine real-time relationships between social activity and mood, fatigue, and pain among older PLWH. Of note, the construct of fatigue in this study is represented by the reported current level of tiredness, consistent with geriatric literature [27]. First, we explored relationships between HIV disease characteristics and social activity (ie, proportion of time alone and number of social interactions per day). Next, we examined concurrent (ie, using responses within the same EMA survey) and lagged (ie, using responses from one survey to predict responses on the next survey) relationships between social activity (ie, alone vs not alone) and sad mood, happy mood, stress, fatigue, and pain level. Regarding concurrent relationships, we hypothesized that being alone would be related to lower ratings of happy mood and higher ratings of sad mood, stress, fatigue, and pain level. Regarding lagged relationships, we hypothesized that being alone on one survey would predict lower ratings of happy mood and higher ratings of sad mood, stress, fatigue, and pain level on the next survey; similarly, we also hypothesized that lower ratings of happy mood and higher ratings of sad mood, stress, fatigue, and pain level on one survey would predict greater likelihood of being alone on the next survey.

## Methods

### Participants

A total of 20 HIV-positive participants aged 51 to 67 years were recruited from ongoing studies at the University of California, San Diego (UCSD) HIV Neurobehavioral Research Program. Inclusion and exclusion criteria are given in Textboxes 1 and 2, respectively. The UCSD Human Research Protections Program approved the study, and all participants completed an assessment of capacity to consent [28] before providing written informed consent.

**Inclusion criteria for this study.**

- HIV seropositive
- Aged 50 years or older at enrollment
- Fluent in English
- Ability to provide written informed consent

**Exclusion criteria for this study.**

- Psychotic disorders (eg, schizophrenia)
- Severe neurological disease (eg, stroke)
- A positive urine toxicology for substances of abuse (any drugs with the exception of marijuana) or breathalyzer test (for alcohol) on the day of testing

### Measures and Procedure

#### HIV Disease Characteristics

To assess HIV disease characteristics, the participants completed a neuromedical examination, consisting of a clinical interview and laboratory testing. HIV serostatus was determined by MedMira Miriad rapid test (Nova Scotia, Canada) or by enzyme-linked immunosorbent assays and confirmatory Western blot test. Nadir CD4 count was self-reported unless the laboratory-tested current CD4 count was lower than the self-reported nadir value; in these cases, the current CD4 count was also recorded as the nadir CD4 count. Plasma HIV viral load was considered undetectable with a lower limit of quantification below 40 copies/mL.

#### Baseline Psychiatric Characteristics

Participants completed the Beck Depression Inventory-II (BDI-II) [29] and the Medical Outcomes Study (MOS) Social Support Survey [30] to assess depressed mood and perceived social support, respectively.

#### Ecological Momentary Assessment Protocol and Procedures

All participants were provided with an Android operating system smartphone. At the initial study visit, the examiner explained how to access and respond to the survey questions on the smartphone. Participants were also given an informational manual (Flesch-Kincaid readability grade level=4.4) on how to use the smartphone and complete the surveys. In addition, participants completed an in-person practice survey monitored by the examiner to assess any difficulties the participant had with the phone or survey questions. On the first day of the study, the examiner called each participant to verify if they had any questions or difficulties completing the surveys at home. If participants were in possession of a personal smartphone, they agreed to carry the study smartphone along with their personal phone for the duration of the study.

Participants completed 5 EMA surveys per day for 1 week, providing up to 35 data points per person. This frequency of assessment has been previously associated with high rates of survey adherence in psychiatric patients [31,32]. Timing of the surveys was set in collaboration with each participant’s preferences and sleep-wake schedules. Time-stamped, de-identified, and encrypted responses were instantly transferred to our password-protected server. At the completion of the EMA assessment period, participants returned the smartphones and completed a follow-up interview regarding their experience carrying and operating the smartphone, as well as their opinion regarding the frequency and duration of the assessments.

#### Ecological Momentary Assessment Survey

Each EMA survey asked participants about their social activity, mood, fatigue, and pain. Social questions included the following:

1. *Who is with you at this moment?* Response choices: alone, spouse or partner, friends, other family members, pets, health care provider, other known people, and unknown people.
2. *Since the last alarm, how many times did you socialize with someone else (eg, spent more than 5 min talking or communicating with someone else)?* To be consistent with literature on the association between social relationships and health outcomes (in which social relationships include only human interactions) [7], participants were identified as being alone when they stated they were currently “alone” or with “pets.”

Mood, fatigue, and pain questions included the following:

1. *How happy do you feel right now?*
2. *How sad do you feel right now?*
3. *How stressed do you feel right now?*
4. *How tired are you right now?*
5. *What is your pain level right now?*

Response choices for happy, sad, stressed, and fatigue were on a 7-point scale from *1=not at all* to *7=extremely*, and response choices for pain were on a 10-point scale from 1*=minimal or no pain* to *10=severe pain*. Regarding the fourth question, we conceptualize fatigue as synonymous with the current level of reported tiredness, consistent with geriatric definitions [27].

### Statistical Analyses

All participants included sufficient EMA data points (>50%) to be included in analyses. Separate mixed-effects regressions were completed to evaluate concurrent (within-survey associations) and lagged (one lag, such that responses from one survey predicted responses on the next survey) associations between dichotomous social activity (alone vs not alone) and levels of mood (happy, sad, and stressed), fatigue, and pain based on the time of day. Specifically, 3 sets of analyses were conducted to examine (1) concurrent associations: social activity as a predictor of same-survey mood, fatigue, and pain; (2) lagged associations: social activity as a predictor of next-survey mood, fatigue, and pain; and (3) lagged associations: mood, fatigue, and pain as predictors of next-survey social activity. For the first set of analyses, 5 linear mixed-effect regression models were used to predict happiness, sadness, stress, fatigue, and pain from the time of day and same-survey social activity. For the second set of analyses, 5 linear mixed-effect regression models were used to predict happiness, sadness, stress, fatigue, and pain from the time of day and previous-survey social activity. For the third set of analyses, 5 mixed-effects logistic regression models were used to predict social activity from the time of day and previous-survey happiness, sadness, stress, fatigue, and pain. In each of the 15 specified mixed-effects regression models (ie, 5 concurrent and 10 lagged models), predictor variables included only time and a single predictor of interest, with the interaction term between time and the predictor of interest included only if significant at *P*<.05.

Missing data were not replaced because mixed-effects regressions are robust to missing data. Notably, the time of day was set as a categorical (5 points per day; Time 1-5) or continuous (hours since midnight) variable based on the results of model comparison using the likelihood ratio test (Multimedia Appendix 1). For this model comparison, we considered 2 different types of models: (1) models in which the effect on the response was linear over time—eg, every 6 h the log odds of social activity would increase by X amount; and (2) models that did not have such a restriction. The choice as to which of the 2 was more appropriate was based on the statistical performance of the models. The linear time effect was preferred for happy mood, sad mood, and pain, whereas the categorical time effect was preferred for stress and fatigue. Thus, for all analyses presented, the time of day was set as a continuous variable for happy mood, sad mood, and pain, whereas the time of day was set as a categorical variable for stress and fatigue. In addition, all models include subject-specific random effects. R software (R Foundation for Statistical Computing, Vienna, Australia), was used for all statistical analyses; the lme4 package for R was used for all mixed-effects regression analyses [33].

## Results

### Relationships Among Social Activity, Demographics, HIV Disease Characteristics, and Baseline Psychiatric Characteristics

The demographic and clinical characteristics of participants are displayed in Table 1. Regarding HIV disease burden, participants were relatively healthy and well controlled, with only one participant not on antiretroviral therapy and having a detectable plasma viral load. Participants completed an average of 86% of EMA surveys (mean 30.3, SD 4.2,), spent an average of 63% of typical awake time alone (SD 31.5; range 9.7-100), and had an average of 1.6 social interactions per day over the one-week study period (SD 0.9; range 0-3.1). Female participants reported being alone significantly less often than male participants (18.2% and 71.9% of surveys on average, respectively; *t*_18_=3.39; *P*=.003; although there were only 3 female participants in the study). HIV disease characteristics were unrelated to the proportion of surveys on which participants reported being alone and to the average number of social interactions per day (Multimedia Appendix 2). In addition, baseline BDI-II scores were unrelated to EMA social activity variables (Multimedia Appendix 2). Higher baseline MOS Social Support scores were significantly related to fewer proportion of surveys on which participants reported being alone (*r*=−.69; *P*<.001) and a greater average number of social interactions per day (*r*=.54; *P*=.02).

### Concurrent Associations: Social Activity as a Predictor of Same-Survey Mood, Fatigue, and Pain

Table 2 displays the linear mixed-effects models for all concurrent associations between social activity (ie, alone vs not alone) and mood, fatigue, and pain. Being alone was related to lower concurrent happiness (beta=−0.300; *P*=.01; Multimedia Appendix 3); however, being alone was unrelated to sadness, stress, fatigue, or pain level. In addition, results showed significant relationships between the time of day and: (1) stress, such that stress was highest at Time 2 (significantly higher than Time 1; *P*=.01) and steadily decreased from Time 2 to Time 5; (2) fatigue, such that fatigue ratings gradually increased over time; and (3) pain level, such that pain levels increased linearly over the course of a day.

### Lagged Associations: Social Activity as a Predictor of Next-Survey Mood, Fatigue, and Pain

**Figure 1:**
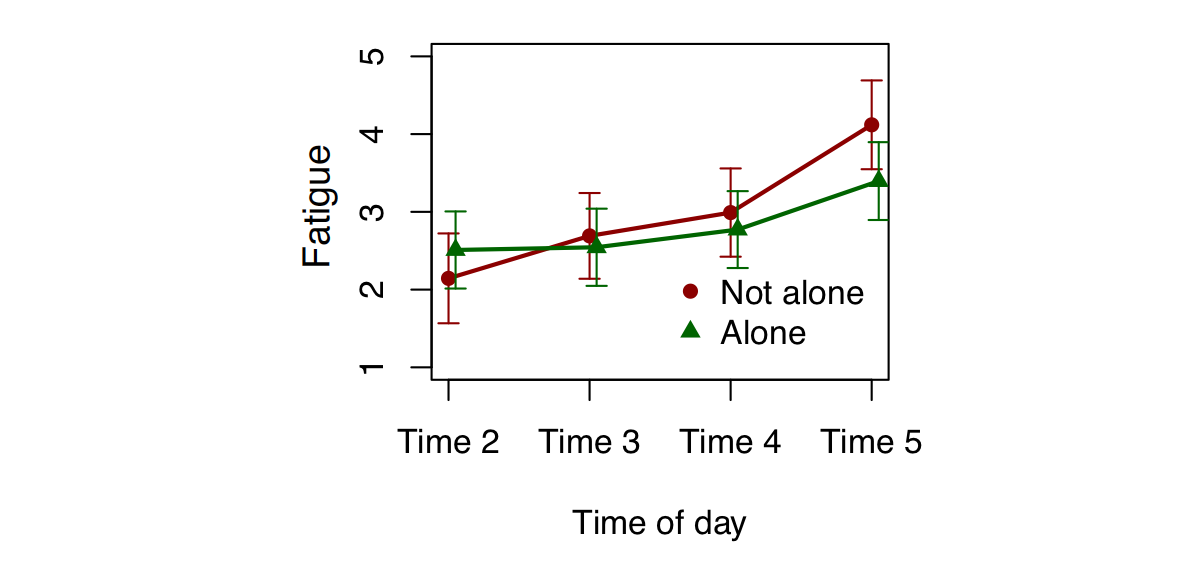
Fatigue at each time point within each day (starting from Time 2) predicted by being alone or not alone on the previous survey. Error bar denotes 95% CI for the mean.

Table 3 displays all linear mixed-effects models in which social activity (ie, alone vs not alone) predicts mood, fatigue, and pain on the next EMA survey. There was a significant interaction between social activity and the time of day on fatigue. The examination of reported fatigue at each time point starting from Time 2 (ie, Time 2-5) showed that being alone at Time 4 was associated with being less tired at Time 5 compared with those who were not alone at Time 4 (beta=−1.089; 95% CI −1.780 to −0.396; *P*=.002; Figure 1). There were no other lagged associations between social activity and mood or pain level.

### Lagged Associations: Mood, Fatigue, and Pain as Predictors of Next-Survey Social Activity

**Figure 2:**
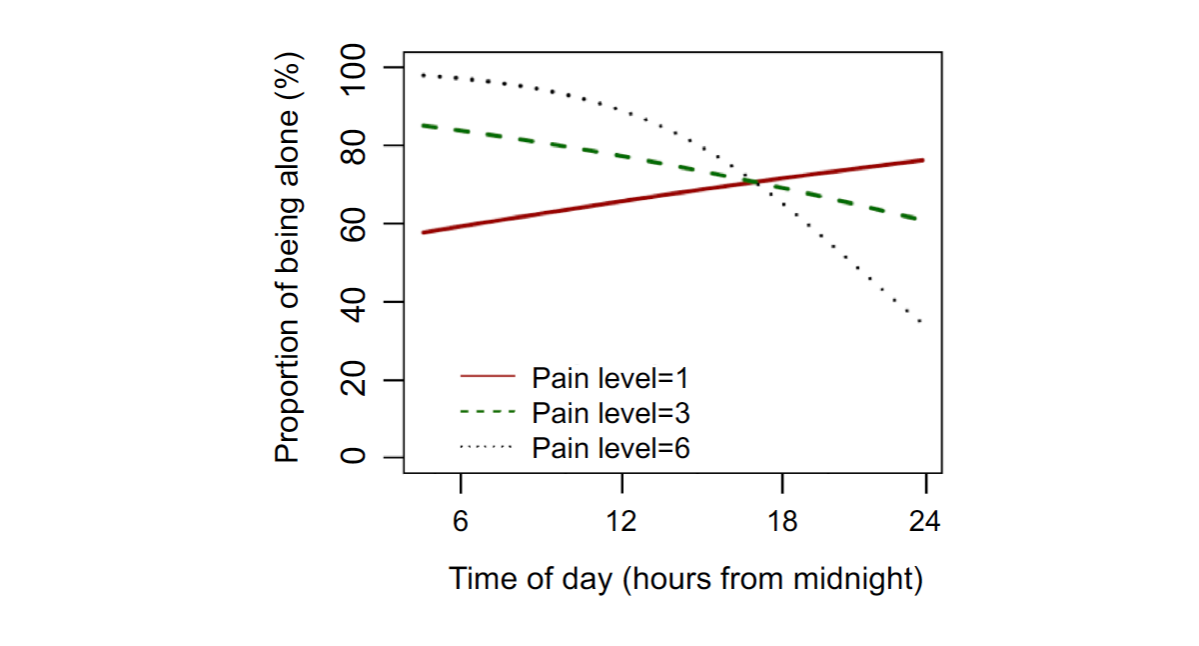
The predicted proportion of surveys on which participants reported being alone over time based on pain levels.

Table 4 shows all mixed-effects logistic regression models in which mood, fatigue, and pain predict social activity (ie, alone vs not alone) on the next EMA survey. There was a significant interaction between pain and the time of day on social activity (beta=0.945; 95% CI 0.901-0.992; *P*=.02). Compared with the lowest pain level, higher pain levels are associated with greater likelihood of being alone earlier in the day and lower likelihood of being alone later in the day (Figure 2). There were no other lagged associations between mood or fatigue and social activity. Notably, results for all concurrent and lagged analyses presented in this report do not differ when the classification of being alone does not include being with “pets.” In addition, a sensitivity analysis removing the 3 female participants from the sample led to similar results and conclusions.

**Table 1:** Demographic and clinical characteristics of participants (N=20).

Characteristics		Value
**Demographic characteristics**		
	Age in years, mean (SD); range	58.8 (4.3); 51-67
	Education in years, mean (SD); range	13.4 (2.7); 8-20
	Sex (male), n (%)	17 (85)
	Race and ethnicity (non-Hispanic white), n (%)	14 (70)
	Employed, n (%)	4 (20)
	Receiving disability (N=11)^a^, n (%)	7 (64)
	Smartphone ownership, n (%)	11 (55)
**HIV disease characteristics^b^**		
	Current CD4 in cell/mL (N=19), median (IQR^c^); range	459 (398-562); 108-750
	Nadir CD4 in cell/mL, median (IQR); range	105 (23.2-205.0); 7-350
	Detectable plasma viral load (N=16), n (%)	1 (6)
	Estimated duration of HIV in years, mean (SD); range	20.4 (7.8); 4.8-29.8
	On ART^d^, n (%)	19 (95)
	History of AIDS (N=18), n (%)	14 (70)
**Baseline psychiatric characteristics**		
	Beck depression inventory-II, median (IQR); range	3.5 (1.0-9.5); 0-38
	MOS^e^ Social Support, mean (SD)	65.5 (33.3); 0-100
**EMA^f^ mood ratings^g^**		
	Happy, mean (SD); range	4.5 (1.1); 1-7
	Sad, mean (SD); range	1.9 (1.1); 1-7
	Stress, mean (SD); range	2.2 (1.2); 1-7
	Fatigue, mean (SD); range	2.7 (0.9); 1-7
	Pain level, mean (SD); range	2.0 (1.4); 1-10

^a^Disability was not assessed in the first 9 participants.

^b^Number of days from collection of neuromedical data to EMA visit: mean 70.9 (SD 151.2).

^c^IQR: interquartile range.

^d^ART: antiretroviral therapy.

^e^MOS: Medical Outcomes Study.

^f^EMA: ecological momentary assessment.

^g^EMA mood ratings are over one week on study.

**Table 2:** Mixed-effects models for associations between social activity and concurrent mood, fatigue, and pain.

Outcome and predictor		Coefficient (95% CI)	*P* value^a^
**Sad mood**			
	Alone	0.078 (−0.140 to 0.299)	.48
	Time (hours)	−0.011 (−0.028 to 0.007)	.24
**Happy mood**			
	Alone	−0.300 (−0.25 to −0.079)	*.008*
	Time (hours)	0.004 (−0.014 to 0.021)	.69
**Stress^b^**			
	Alone	−0.064 (−0.268 to 0.141)	.54
	Time 2	0.300 (0.068 to 0.532)	*.01*
	Time 3	0.227 (−0.009 to 0.463)	.06
	Time 4	0.155 (−0.083 to 0.393)	.20
	Time 5	−0.152 (−0.391 to 0.088)	.22
**Fatigue^b^**			
	Alone	0.122 (−0.148 to 0.392)	.38
	Time 2	0.260 (−0.054 to 0.574)	.11
	Time 3	0.443 (0.126 to 0.760)	*.007*
	Time 4	0.703 (0.383 to 1.024)	*<.001*
	Time 5	1.520 (1.196 to 1.844)	*<.001*
**Pain**			
	Alone	0.035 (−0.161 to 0.231)	.73
	Time (hours)	0.021 (0.005 to 0.036)	*.01*

^a^Italics indicate *P*<.05.

^b^Models for stress and fatigue use time as a categorical variable; coefficient values for Time 2 to Time 5 indicate the change in the outcome variable compared with Time 1.

**Table 3:** Mixed-effects models for associations between social activity and next-survey mood, fatigue, and pain.

Outcome and predictor		Coefficient (95% CI)	*P* value^a^
**Sad mood**			
	Alone	0.102 (−0.141 to 0.348)	.41
	Time (hours)	−0.012 (−0.037 to 0.013)	.24
**Happy mood**			
	Alone	−0.034 (−0.301 to 0.226)	.80
	Time (hours)	0.011 (−0.016 to 0.038)	.43
**Stress^b^**			
	Alone	0.008 (−0.230 to 0.250)	.95
	Time 3	−0.100 (−0.353 to 0.154)	.44
	Time 4	−0.170 (−0.425 to 0.085)	.19
	Time 5	−0.502 (−0.757 to −0.247)	*<.001*
**Fatigue^b^**			
	Alone	0.365 (−0.169 to 0.898)	.18
	Time 3	0.546 (0.008 to 1.083)	*.048*
	Time 4	0.846 (0.296 to 1.395)	*.003*
	Time 5	1.975 (1.427 to 2.523)	*<*.*001*
	Alone × Time 3	−0.511 (−1.196 to 0.176)	.15
	Alone × Time 4	−0.584 (−1.273 to 0.110)	.10
	Alone × Time 5	−1.089 (−1.780 to −0.396)	*.002*
**Pain**			
	Alone	−0.016 (−0.240 to 0.206)	.89
	Time (hours)	0.027 (0.010 to 0.050)	*.02*

^a^Italics indicate *P*<.05.

^b^Models for stress and fatigue use time as a categorical variable; coefficient values for Time 2 to Time 5 indicate the change in the outcome variable compared with Time 1.

**Table 4:** Mixed-effects logistic regression models for associations between mood, fatigue, and pain and next-survey social activity.

Outcome (social engagement) and predictor			Odds ratio (95% CI)		*P* value^a^
**Model 1**					
	Sad mood	1.017 (0.799 to 1.294)		.89	
	Time (hours)	1.002 (0.934 to 1.074)		.96	
**Model 2**					
	Happy mood	0.904 (0.713 to 1.147)		.41	
	Time (hours)	1.007 (0.938 to 1.080)		.85	
**Model 3**					
	Stress^b^	0.858 (0.665 to 1.107)		.24	
	Time 3	1.091 (0.535 to 2.223)		.81	
	Time 4	0.935 (0.456 to 1.917)		.86	
	Time 5	0.952 (0.463 to 1.959)		.89	
**Model 4**					
	Fatigue^b^	1.064 (0.872 to 1.298)		.54	
	Time 3	1.01 (0.501 to 2.037)		.98	
	Time 4	0.771 (0.381 to 1.561)		.47	
	Time 5	0.854 (0.413 to 1.769)		.67	
**Model 5**					
	Pain	2.647 (1.092 to 6.412)		*.03*	
	Time (hours)	1.107 (0.991 to 1.235)		.07	
	Pain × Time	0.945 (0.901 to 0.992)		*.02*	

^a^Italics indicate *P*<.05.

^b^Models for stress and fatigue use time as a categorical variable; coefficient values for Time 2 to Time 5 indicate the change in the outcome variable compared with Time 1.

## Discussion

Examining real-time, immediate predictors of social activity is an important and novel approach to further our understanding of social experiences of older PLWH. Specifically, our findings provide a framework for understanding social activity in the context of mood, fatigue, and pain among older PLWH. This study found being alone was associated with lower concurrent happiness and later-day fatigue, and higher pain levels predicted greater likelihood of being alone earlier in the day and lower likelihood of being alone later in the day (compared with lower pain levels). Simply stated, although being with others was related to increases in fatigue and pain throughout the day, people were happier when they were with others than when they were alone.

The importance of the association between more social activity and greater happiness warrants a thorough discussion. This finding is critical for our understanding of psychological well-being of older PLWH. Although seemingly contradictory, our finding that participants were happier when they were with others (compared with when they were alone) even though pain and fatigue increased is supported by neurobiological and behavioral medicine literature. Neurobiologically, positive social interactions induce activity in the mesocorticolimbic (ie, reward) system and are experienced as pleasurable [34]; thus, social activity is likely reinforcing beyond potentially limiting factors such as pain or fatigue. Our finding is also consistent with the “stress buffering” theoretical model such that social relationships may “buffer” the adverse effects of a stressor (eg, pain) on health outcomes (eg, mood) [35]. Furthermore, happiness has been associated with a variety of positive health and lifestyle factors among older adults, including physical activity, competence, functional capacity, disease management, quality of social network, and general life satisfaction [36,37]. These positive health and lifestyle factors are critical for maintaining optimal quality of life among older adults, especially those living with a chronic disease such as HIV [38]. Thus, our findings support further examination of the benefits of social activity on real-time happiness and psychological well-being, and whether increased social activity may have longer-term effects on happiness. On the basis of the results of this study and existing literature on associations of overall health and happiness, the immediate positive influence of increased social activity on happiness has potential to improve many aspects of an older adult’s quality of life.

Although there were no significant associations between social activity and concurrent sadness or stress, this is likely because our participants reported overall low levels of sadness and stress with little variability compared with reported levels of happiness. Possible interpretations for this finding include the following: (1) our sample may not be very representative of older PLWH among whom depressive symptoms and stress are common, (2) our measures of sadness and stress may not be sensitive enough for this population, or (3) our measure of positive affect (happiness) may be a more sensitive indicator of mood and well-being in older PLWH with minimal-to-no depressive symptoms (as indicated by baseline BDI-II scores). Future research is needed to understand the clinical relevance of fluctuations in EMA-measured happiness among older PLWH without clinically elevated depressive symptoms or mood disorders, and whether consistently low happiness ratings in the context of decreased social activity or social isolation may predict feelings of sadness, loneliness, or onset of a depressive episode.

In contrast to our finding regarding increased happiness with social activity, results also showed that spending time with others predicted being more fatigued on the next survey. The relationship between social activity and fatigue is important to consider in the context of the association between social activity and happiness, and provides preliminary evidence to support potential clinical interventions promoting social activity [39-41] while simultaneously improving our understanding of strategies that help older PLWH manage fatigue in an effort to improve happiness and psychological well-being in this population. Notably, lagged analyses also elucidated an interaction effect between pain and time of day on later social activity. Participants reporting higher pain levels were more likely to be alone in the morning and less likely to be alone in the evening and night compared with those reporting lower pain levels. Although we are unable to determine reasons for this relationship with our available data, results show that participants in our sample of older PLWH were engaging in evening social activities despite experiencing high levels of pain. Further examination of this pattern is needed to determine whether this may be evidence of the ability to utilize effective coping techniques (eg, social support) when needed among older PLWH.

As previously reported by Moore et al [19], our sample of PLWH aged 50 years and older reported being alone for a majority of participants’ typical waking hours. This is considerably higher than results reported by the national American Time Use Survey in which adults aged 65 years and older reported spending 23% to 42% of waking, nonwork time alone [42]. Although our results may be influenced by the high rate of unemployment in our sample, our results are consistent with the previous research showing that older PLWH are at an increased risk for reduced social activity [8-10]. Future research on social activity must not only characterize levels of social activity but must also focus on understanding and disentangling different reasons for decreased social activity within different clinical populations, with the ultimate goal of developing targeted interventions.

Results also showed that HIV disease characteristics (ie, current and nadir CD4 counts, detectable plasma viral load, estimated duration of HIV, antiretroviral therapy status, and history of AIDS) were unrelated to the proportion of time spent alone and the average number of reported social interactions per day. Although previous research links social isolation to accelerated HIV disease progression [20], this is often in the context of perceived loneliness and social support. In contrast, the current EMA social activity data capture a more objective level of overall social activity. Findings from this study warrant further examination of the association between HIV disease progression and levels of social activity. Furthermore, participants who reported more social support at baseline (via the MOS Social Support survey) demonstrated a lower proportion of time alone and a higher number of daily social interactions over the 1-week EMA period. This finding also supports the convergent validity of our EMA social activity measures, and suggests that participants’ perceptions of support received from their social networks may be accurately related to their average levels of social activity. These relationships between social support and EMA-assessed social activity were expected, as social support often relates to the number of social contacts and frequency of social interactions across many clinical and nonclinical populations [43]. Finally, we found that females reported spending less time alone compared with males. Because we cannot make any definitive conclusions from a sample that only includes 3 women, further exploration of possible sex differences in social activity and health outcomes is needed among older PLWH in future studies.

This study is strengthened by its use of real-time data; however, there are several limitations. First, the relatively small sample size limits generalizability and statistical power to detect associations between social activity and baseline, laboratory-measured clinical characteristics. Furthermore, our sample was relatively healthy and had little-to-no HIV disease burden, which may not be widely representative of the general population of older PLWH in the United States. Also of note, the majority of participants in our sample were unemployed. Because employment is an activity that often engenders social activity (eg, interactions with coworkers and increases financial resources), our results may not be generalized to older PLWH who are employed; however, our sample also represents a real-world sample of older PLWH among whom unemployment is very common [44]. Future studies would benefit from further understanding associations between reasons for unemployment and levels of social activity. Next, because the data came from a small pilot study with the aim of assessing overall daily functioning in older PLWH, there were no data collected on other specific factors related to social isolation, such as perceived loneliness, reasons for being alone (eg, preference vs access to social activities), or structural social factors (eg, marital or relationship status). Relatedly, future research may benefit from understanding the real-time impact of pet ownership and time spent with pets on mood and physical health. In addition, the EMA item related to being alone or with others may not be the best representation of social isolation because it only allows us to capture participants’ social activity at the exact moment of the survey administration—that is, if participants were alone when completing the survey but were with others right up until the survey completion time, then they will report being alone although they may not be socially isolated. Future studies may consider using mobile sensing technologies and global positioning system data to better characterize social activity [45]. Assessing real-time associations between social activity and markers of mental and physical health, as we have done in this report, also lays the groundwork for developing potential real-time interventions for improving happiness, psychological well-being, and overall quality of life.

Overall, this study provides preliminary evidence for the real-time associations between social activity and mood, fatigue, and pain in older PLWH. Our findings showed that spending time with others was associated with increased happiness. The results of this study warrant additional research on the potential benefits of social activity-based interventions for improving well-being among older PLWH despite presence of fatigue or pain. Furthermore, with mounting evidence showing that older PLWH spend a great deal of time alone, future research must continue to characterize social activity as well as explore causes for and consequences of limited social activity among this population.

## Appendix

Multimedia Appendix 1 Comparisons between mixed-effects models with continuous time and mixed-effects models with categorical time.

Multimedia Appendix 2 Relationships between social activity, demographics, HIV disease characteristics, and baseline psychiatric characteristics.

Multimedia Appendix 3 Average happiness ratings by social activity (alone vs not alone) within participants. Error bar denotes 95% CI for the mean.

## Abbreviations

BDI-II: Beck Depression Inventory-II
EMA: ecological momentary assessment
MOS: Medical Outcomes Study
PLWH: persons living with HIV
UCSD: University of California, San Diego
