# Supplementary material for: Temporal Associations Between Social Activity and Mood, Fatigue, and Pain in Older Adults With HIV: An Ecological Momentary Assessment Study
Source: JMIR Ment Health. 2018 May 14;5(2):e38. doi: 10.2196/mental.9802 (PMC5972192; doi:10.2196/mental.9802)
Supplement: Multimedia Appendix 1 [file mental_v5i2e38_app1.pdf]

Mixed-effects model comparison	AIC	Log Likelihood	deviance	Chi-square	DF	p-value
Sad						
Continuous time	1650	-821.236	1642	0.457	3	0.93
Categorical time	1656	-821.007	1642			
Happy						
Continuous time	1663	-827.387	1655	0.587	3	0.90
Categorical time	1663	-827.094	1654			
Stress						
Continuous time	1599	-795.281	1591	15.3	3	0.002
Categorical time	1589	-787.623	1575			
Fatigue						
Continuous time	1972	-982.231	1964	12.8	3	0.005
Categorical time	1966	-975.813	1952			
Pain						
Continuous time	1616	-804.168	1608	4.259	3	0.23
Categorical time	1618	-802.039	1604			
