# Supplementary material for: Temporal Associations Between Social Activity and Mood, Fatigue, and Pain in Older Adults With HIV: An Ecological Momentary Assessment Study
Source: JMIR Ment Health. 2018 May 14;5(2):e38. doi: 10.2196/mental.9802 (PMC5972192; doi:10.2196/mental.9802)
Supplement: Multimedia Appendix 2 [file mental_v5i2e38_app2.pdf]

	Relationship to proportion of time alone	p-value	Relationship to average social interactions per day	p-value
**Demographics**				
Age (yrs)	-.03	.90	.14	.58
Education (yrs)	-.01	.96	.23	.35
Sex (male vs. female)	.62	**.003**[b]	-.25	.34
Race/Ethnicity (White vs. non-White)	.12	.60	.10	.71
Employed (yes vs. no)	.07	.76	-.23	.37
Receiving Disability (yes vs. no)[a]	.25	.45	-.25	.48
Smartphone Ownership (yes vs. no)	-.27	.35	.26	.34
**HIV Disease Characteristics**				
Current CD4 (cell/μl)	-.09	.73	-.03	.90
Nadir CD4 (cell/μl)	.04	.88	-.23	.35
Estimated duration of HIV (yrs)	.18	.45	.27	.28
History of AIDS (yes vs. no)	-.15	.53	.40	.11
**Baseline Psychiatric Characteristics**				
Beck Depression Inventory-II	.13	.58	.29	.24
MOS-Social Support	-.69	**<.001**	.55	**.02**

Note. Relationships are displayed as Pearson r values; for dichotomous variables, independent t-tests were conducted and t-scores were converted to r values; social activity variables were not compared by HIV plasma viral load detectability or antiretroviral therapy (ART) status because only one participant had detectable plasma viral load and was not on ART

[a]n=11; disability was not assessed in the first nine participants

[b]Males had a higher proportion of time alone than females
