# Supplementary figures and images for: Temporal Associations Between Social Activity and Mood, Fatigue, and Pain in Older Adults With HIV: An Ecological Momentary Assessment Study
Source: JMIR Ment Health. 2018 May 14;5(2):e38. doi: 10.2196/mental.9802 (PMC5972192; doi:10.2196/mental.9802)

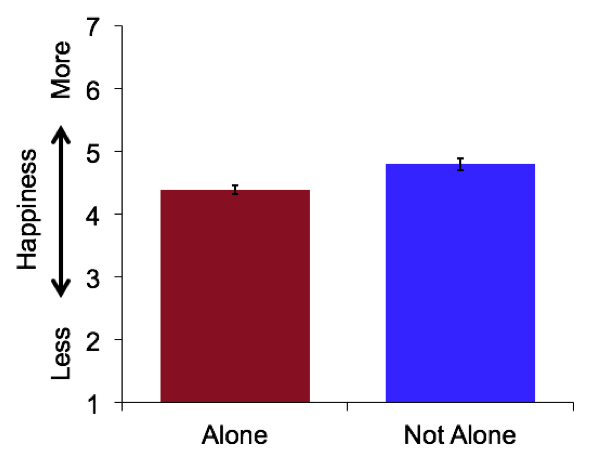

Supplement: Multimedia Appendix 3 [file mental_v5i2e38_app3.png]
